# Determination of Structural Factors Contributing to Protection of Zinc Fingers in Estrogen Receptor α through Molecular Dynamic Simulations

**DOI:** 10.1021/acs.jpcb.4c05730

**Published:** 2025-02-12

**Authors:** Patricia B. Lutz, Wesley R. Coombs, Craig A. Bayse

**Affiliations:** † Department of Science & Mathematics, Regent University, Virginia Beach, Virginia 23464, United States; ‡ Department of Chemistry and Biochemistry, Old Dominion University, Norfolk, Virginia 23529, United States

## Abstract

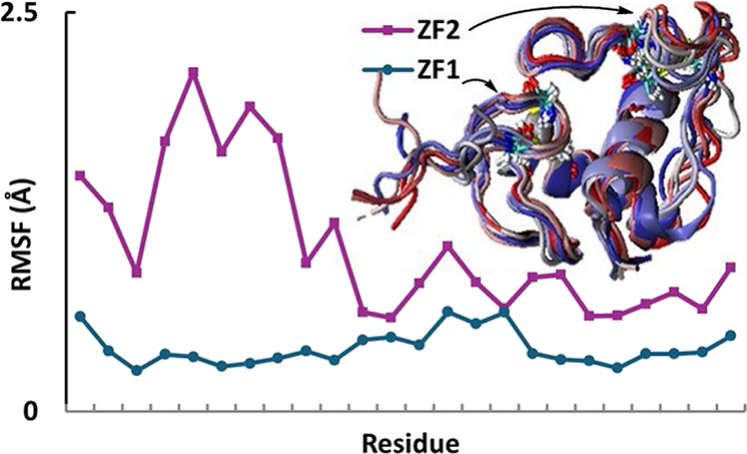

The ERα transcription factor that induces tumor growth is a potential target for breast cancer treatment. Each monomer of the ERα DNA-binding domain (ERαDBD) homodimer has two conserved (Cys)_4_-type zinc fingers, ZF1 (N-terminal) and ZF2 (C-terminal). Electrophilic agents release Zn^2+^ by oxidizing the coordinating Cys of the more labile ZF2 to inhibit dimerization and DNA binding. Microsecond-length molecular dynamics (MD) simulations show that greater flexibility of ZF2 in the ERαDBD monomer leaves its Cys more solvent accessible and less shielded from electrophilic attack by sulfur-centered hydrogen bonds than ZF1 which is buried in the protein. In the unreactive DNA-bound dimer, the formation of the dimer interface between the highly flexible D-box motif of ZF2 decreases the solvent accessibility of its Cys toward electrophiles and increases the populations of sulfur-containing hydrogen bonds that reduce their nucleophilicity. Examination of these factors in ERαDBD and other proteins with labile ZF motifs may reveal new targets to treat viral infections and cancer.

## Introduction

Zinc fingers (ZFs) are small protein domains with Zn^2+^ tetrahedrally coordinated to at least 2 Cys and His.^1^ ZFs are found in three classes: (1) CCHH, (2) CCCH, and (3) CCCC. ZFs are involved with many cellular processes, including replication, repair, transcription, translation, cell proliferation, apoptosis, metabolism, and signaling.^2^ Three percent of the human genome encodes for these small (20–60 residue) ZF domains, the majority of which are transcription factors.^3^ Zinc coordination maintains the correct tertiary structure of the protein for recognition and binding. Zn^2+^ itself is redox inactive, but oxidation of the Cys thiolates releases Zn^2+^, causing the ZF to lose its tertiary structure and function.^4−10^ ZF reactivity increases with the number of Cys (CCHH^0^ < CCCH^1–^ < and CCCC^2–^).^11−13^ For example, a CCHH ZF was oxidized by H_2_O_2_ more than 200 times slower than a CCCC ZF.^14^ Labile Zn-bound thiolates can react with electrophilic agents, making them attractive drug targets.^15,16^

Reactivity differences in ZF proteins of the same class can be attributed to the environment of the surrounding protein. Elucidating the factors determining Cys lability is important for drug discovery and include (a) the Zn^2+^ coordination sphere;^11,17^ (b) interactions between the Zn^2+^ coordination sphere and the surrounding protein;^17−19^ (c) protein flexibility;^20^ (d) solvent accessibility to the Cys residues;^11,21^ and (e) nucleic acid-binding.^22,23^ Of these, electrostatic and steric shielding by the protein are considered to be the most essential factors in determining the reactivity of a Zn^2+^ bound thiolate.^18,24−27^ In particular, sulfur-containing hydrogen bonds (SCHBs) with backbone N–H groups or positively charged Arg or Lys residues can protect Zn-bound thiolates by reducing their susceptibility to electrophilic attack by 1–2 orders of magnitude.^19,24−26,28−31^ Conformational flexibility may also play an important role by modulating the H-bond network that protect the Zn^2+^ coordination sphere.^32,33^ Although many studies have examined the lability of ZFs, the relative reactivity of proteins with multiple ZF motifs remains under investigation.

**Figure 1:**
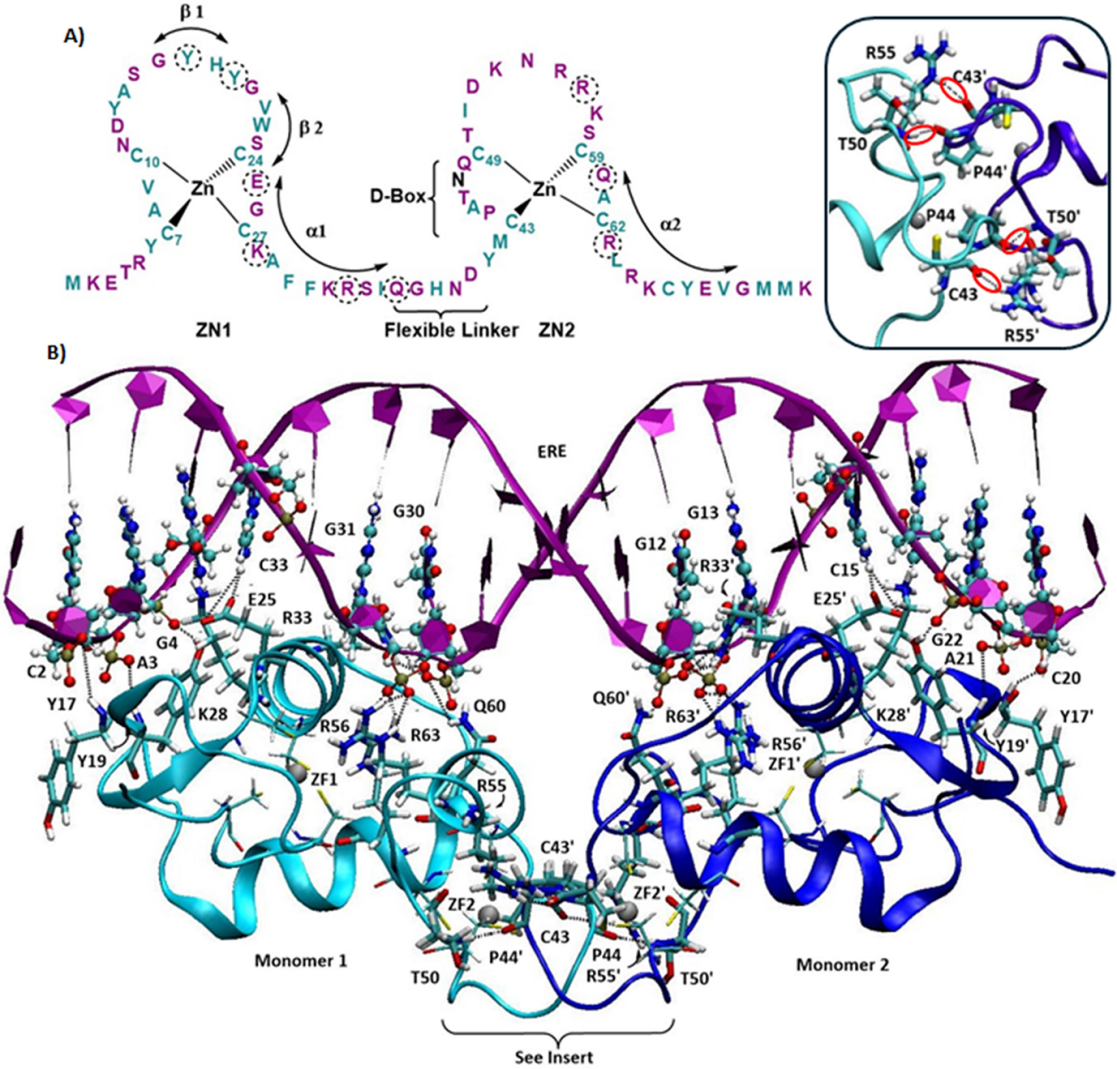
Structure of ERαDBD and the dimer bound to the estrogen response element (ERE). (A) Amino acid sequence of ERαDBD. DNA binding residues are circled with flexible and rigid residues in purple and teal, respectively. (B) X-ray structure of ERαDBD_2_ in complex with ERE (PDB: 1HCQ(34)). DNA bases are modeled with CPK, residues with licorice, and Zn^2+^ with VDW. Monomer 2 residues are designated with a prime (′). The inset above the figure is a magnified view of the dimer interface, rotated 90° with key hydrogen bonds circled in red, created using VMD and ChemDrawPro 14.

The human estrogen receptor alpha transcription factor DNA binding domain (ERαDBD), found in 70% of breast cancer tumors,^34,35^ has two conserved CCCC zinc-finger proteins, ZF1 (residues 7–27) and ZF2 (residues 43–62) (Figure 1A).^36,37^ ERαDBD contains antiparallel β-sheets, β_1_ (G16-H18), β_2_ (V21-C24) and two major α-helical regions (α_1_ (E25-Q36) and α_2_ (Q60-V70)) connected by a linker sequence (G37-D42), and the D-box loop (C43-C49) that forms the dimer interface. An additional unstructured loop is found between C49 and C59 of ZF2. ERαDBD is monomeric in solution and binds cooperatively to DNA to form a dimer. Binding of one monomer to the estrogen response element (ERE) half-site enhances binding in the second half-site, with the D-box sequence (P44-Q48) responsible for dimerization.^38^ In the DNA-bound dimer (ERαDBD_2_-ERE), ZF1 interacts with DNA while ZF2 stabilizes the dimer (Figure 1B).^39−41^ The overexpression of ERα induces tumor growth,^42,43^ making it a target for breast cancer treatment.^35,44^ However, some ER-positive breast cancer cells are resistant to Tamoxifen (TAM), a popular antihormone breast cancer drug that binds to and inhibits ERα.^45,46^ The electrophilic agents 2,2′-dithiobenzamide (DIBA) and benzisothiazolone (BITA) inhibited tumor growth in ERα ZFs, including TAM-resistant cells, without affecting other nuclear receptors and zinc proteins.^47,48^ These reducible sulfur-containing oxidizing agents, attack the Zn-bound Cys to form either internal cystine bonds or external disulfide bonds with the electrophile.^12,49,50^ In either case, the oxidized Cys can no longer effectively coordinate to the metal ion, leading to Zn^2+^ release and loss of structure and function.^10^ The nature of the oxidation will depend upon the electrophilic agent and the ZF motif.^12,13,17,49,51^ While ZF1 was not susceptible to redox agents,^27^ Zn^2+^ was preferentially ejected from ZF2 to prevent dimerization and DNA binding.^41,47^ The greater reactivity of ZF2 has been attributed to a lower p*K*_a_ due to the larger number of basic residues compared to ZF1.^27^ DNA binding also protects the ERαDBD dimer from oxidation relative to the free monomer.^41^ Similarly, the C-terminal ZF of HIV-1 drug target NCp7’s is eight times more reactive than the N-terminal of its two CCCH ZFs.^52,53^ In contrast, the N-terminal ZF of Fog-1, involved in myeloproliferative disorders, is preferentially oxidized.^22,54^ Similarly, the reducible sulfur and selenium compounds disulfiram and ebselen effectively inhibited SARS-CoV-2 by releasing Zn^2+^ from the ZF-containing proteins nsp10, nsp13, and nsp14.^8,55^ Understanding the factors that control the lability of Zn^2+^ in ZF proteins could help design drug therapies for various viruses and cancers.

To better understand the factors modulating thiolate reactivity, molecular dynamics (M.D.) simulations were used to examine the variable reactivity of ZF1 and ZF2 in monomeric ERαDBD, the free dimer (ERαDBD_2_), and ERαDBD_2_-ERE. MD simulations are an indispensable tool for analyzing protein flexibility.^56^ Previous MD studies that compared the structure and dynamics of the ZF2 region of ERαDBD to the glucocorticoid receptor DNA-binding domain (GRDBD)^57^ and of the binding of the DNA to the ERαDBD dimer^58,59^ found the linker and the D-box in ERαDBD to be the most flexible regions; those regions became ordered and had lower solvent accessibility when complexed to the DNA in ERαDBD_2_-ERE.^57,59^ An analysis of microsecond-length simulations show that this change in flexibility also affects the population of SCHBs and the solvent accessibility of the Cys, which can be linked to protection from electrophilic attack.

## Methods

The initial protein and protein–DNA models were obtained from the crystal structure of ERαDBD_2_-ERE (PDB: 1HCQ).^39^ H18 and H38 were assigned as neutral histidine residues based upon processing through the H++ server.^60^ Zn^2+^-coordinated Cys were modeled as cysteinates as these residues are generally considered to be fully deprotonated in CCCC ZF motifs.^61,62^ The biomolecules were modeled in AMBER 22^63^ with the FF14SB^64^ and OL15^65^ force fields for the protein and DNA, respectively. The bonded zinc AMBER force field (ZAFF),^66^ shown to be a reliable model, was used for the Zn^2+^ coordination sites.^67^ All systems were solvated with an 11.0 Å box of TIP3P water.^68^ The monomer was neutralized with Cl^–^ ions, and the DNA-bound dimer was neutralized with Na^+^ without including additional ions to buffer the system, which could lead to an overestimation of the electrostatics. SHAKE constraints were applied to bonds to hydrogen atoms.^69^ Models were initially heated and equilibrated with constant volume and temperature from 0 to 300 K using Langevin dynamics. The models were then equilibrated within the NPT ensemble, followed by microsecond-length production simulations, with three independent trajectories for the monomer ERαDBD, two for the DNA-bound dimer ERαDBD_2_-ERE, and three for the free dimer, ERαDBD_2_. The data was averaged over the runs. For ERαDBD_2_ and ERαDBD_2_-ERE, the two monomers in the dimers were averaged together. The AMBER utility CPPTRAJ^70^ was used to calculate the root-mean-square deviation (RMSD) (SI Figure S1) and root-mean-square fluctuation of the Cα (RMSF) of individual residues and averaged over the one-microsecond trials. The solvent-accessible surface area (SASA) for each Zn^2+^ bound Cys S was calculated using the linear combinations of pairwise overlaps (LCPO) algorithm of Weiser et al.^71^ CPPTRAJ was also used for hydrogen bond analysis with donor–acceptor cutoff distances and angles of 3.3 Å (O/N H-bonds) and 4.0 Å (SCHBs) and angles of 135° (O/N H-bonds) and 90.0° (SCHBs), respectively. Thresholds for SCHBs are similar to those in previous studies.^72−74^ The Visual Molecular Dynamics (VMD)^75^ program was used to visualize and analyze all trajectories.

## Results and Discussion

Microsecond MD simulations of ERαDBD, ERαDBD_2_, and ERαDBD_2_-ERE were conducted to determine the effects of flexibility and SCHB on shielding of the ZF Cys. The face-to-face ERαDBDs each bind to a palindromic half-site composed of six base pairs on the ERE as they dimerize (Figure 1B). The recognition helix (C24-Q36) binds to the DNA major groove through residues E25, K28, K32, and R33.^39,76^ Other H-bonds and salt bridges found between Y17, Y19, R56, and R63 and the ERE phosphate backbone were in agreement with those found in the crystal structure. The dimer interface consists of backbone–backbone NH···OC H-bonds between the T50-P44′, T50′-P44, C43-R55′, and R55-C43′ residues.^39,57,59^ Populations of other H-bonds are comparable for each monomer and in general agreement with previous MD studies (Figure 1B, SI Tables S1, S2, S3, and Figure S2).^39,57^

### Flexibility of the Free ERαDBD Monomer

**Figure 2:**
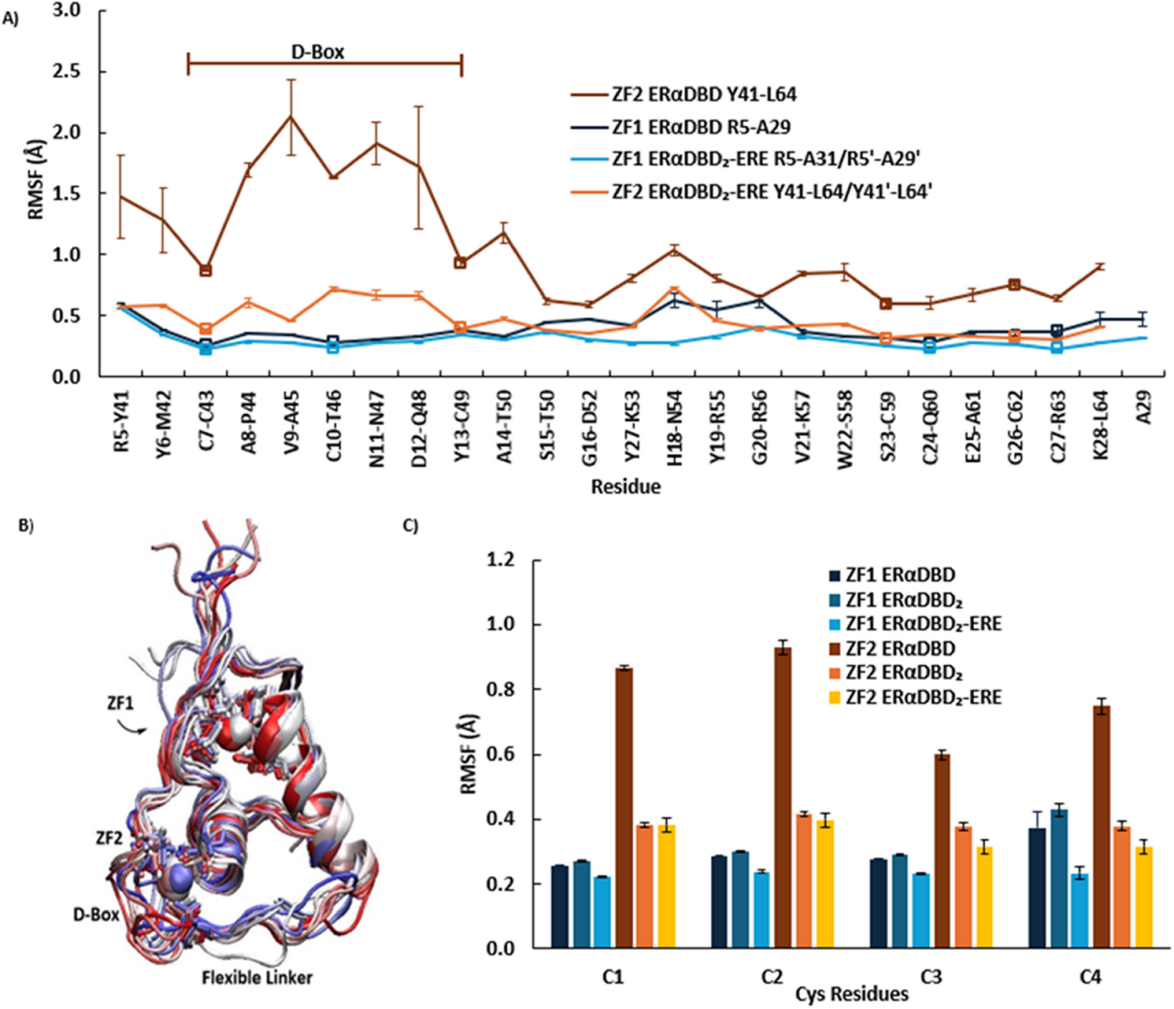
(A) RMSF for ZF1 and ZF2 of free ERαDBD and the DNA-bound dimer ERαDBD_2_-ERE. The Zn^2+^ bound Cys are boxed. The RMSF data for the dimer was averaged over the two monomers, with standard error bars. (B) Snapshots of ERDBD monomer changing from red to white to blue over the course of the simulation, display the flexible nature of the D-Box and Linker. (C) RMSF for ZF Cys residues {ZF1: C1 (C7), C2 (C10), C3 (C24), C4 (C27)}; {ZF2: C1 (C43), C2 (C49), C3 (C59), C4 (C62)} for free ERαDBD, the dimer alone ERαDBD_2_ and the DNA-bound dimer ERαDBD_2_-ERE, with standard error bars.

Various studies have shown that ZF2 of the ERαDBD monomer is more reactive to electrophilic agents than ZF1.^24,27,41,47^ An increased conformational flexibility of ZFs correlates with increased reactivity with electrophiles.^14^ Based upon B-factors obtained from X-ray crystallographic data, residues have been classified as either flexible (GTRSNQDPEK) or rigid (WYFCIVHLMA), with the flexibility of a sequence increasing related to the number of flexible residues.^77^ ZF1, nine of the 17 non-Cys residues are classified as rigid, whereas 13 of ZF2’s 16 non-Cys residues are flexible. ZF1 is less dynamic over two trials relative to ZF2 (RMSF average of 0.4 Å versus 1.0 Å, Figure 2A). The flexibility of C1 through C4 in ZF2 is greater than in ZF1, even though they are tethered to the Zn^2+^ ion (Figure 2C). Independent-sample *t* tests indicate that RMSF differences between ZF1 and ZF2 are statistically significant (*p* < 0.001). The flexibility of the ZF2 C49–C59 and D-box loops in MD simulations is in agreement with their low definition in the NMR study and high B-factors in the X-ray crystal structure.^38,57,76^ NMR data describe T4-C24 part of (ZF1) as well-defined but Q36-S58, which includes the linker, 37–43, the D-Box 43–49, and the unstructured loop at the top of ZF2 between C49 and C59, to be poorly defined.^38^ From X-ray crystallography, H38, N39, and D40, which are members of the five-residue linker from Q36 to D40, NH protons were in rapid exchange in the NMR structure, had high-temperature factors in the crystal, indicating a lack of order. C43–C49 (D-Box), where the monomers dimerize, is the least defined section of the solution structure, suggesting that it is flexible.^76^ The snapshots of ERαDBD monomer over the simulation display the flexible nature of the D-Box and Linker (Figure 2B). The dynamic nature of ZF2, may contribute to the overall higher reactivity of free ERαDBD with electrophiles, noting that C59, with the smallest RMSF, is less reactive than C62 in a metal-free peptide model of ZF2.^27^

### Flexibility of the DNA-Bound ERαDBD Dimer

The ERαDBD monomers dimerize when bound to DNA (Figure 1B), which protects the ZFs from oxidation, even at high oxidant concentrations.^41^ Consistent with a previous MD study, the flexibility of the individual ERαDBD monomers was considerably reduced in simulations of ERαDBD_2_-ERE (SI Figure S1).^57^ NMR studies also show that the flexible regions of the monomer become more structured when ERαDBD has dimerized and bound to DNA.^76^ The average RMSF of ZF1 and ZF2 decreased by 24 and 58%, respectively, due to stabilization by the protein–DNA interactions and the intermolecular backbone–backbone H-bonds between the D-box T50-P44′ and T50′-P44 residues (Figure 2A). In particular, the Zn^2+^ bound Cys RMSF values were reduced by 25% for ZF1 and 56% for ZF2 (Figure 2 C). The RMSF differences between the monomer and DNA-bound dimer ZF2 Cys residues were statistically significant (*p* < 0.001).

**Figure 3:**
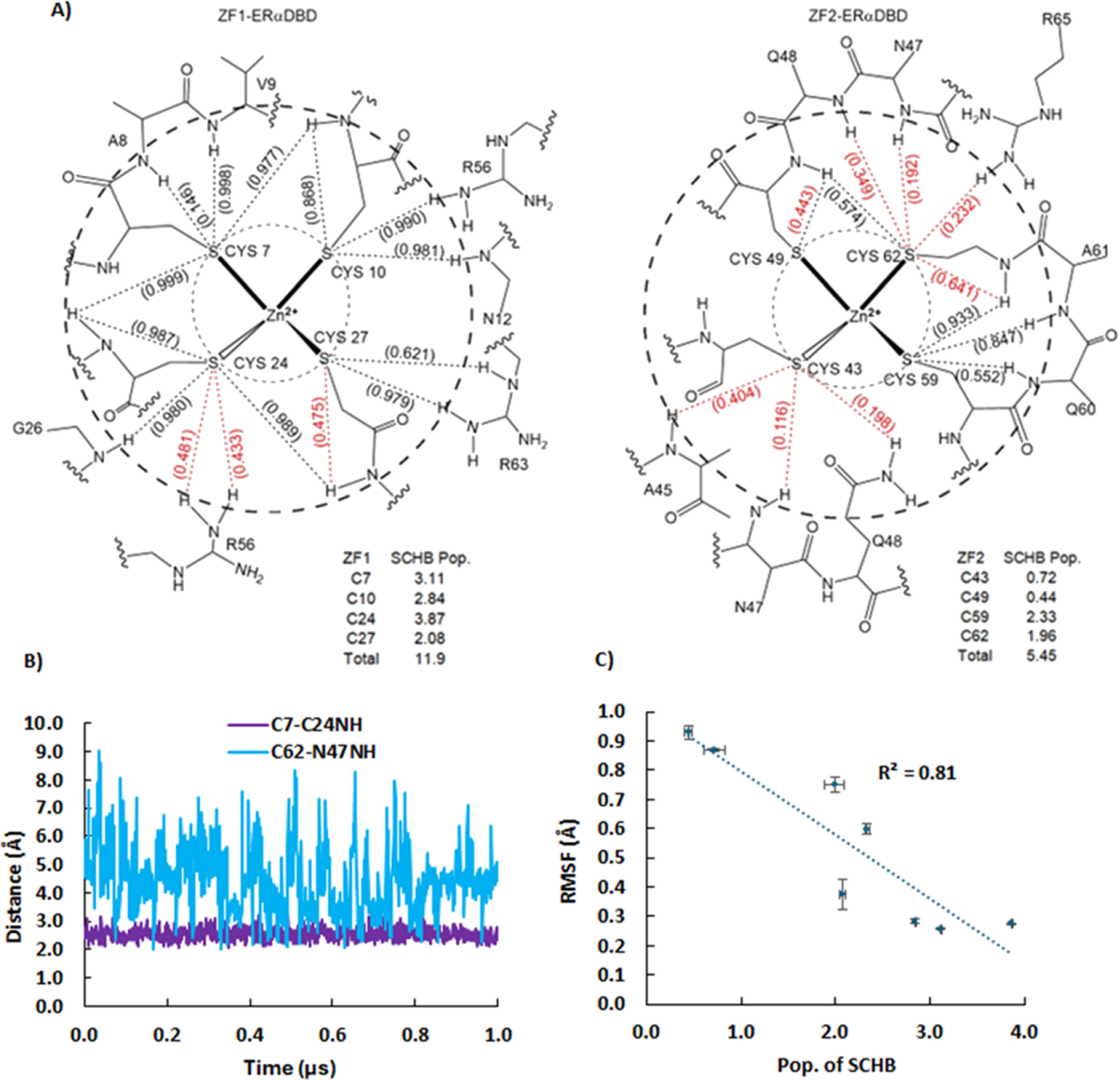
(A) Monomer SCHBs of the inner and outer coordination sphere of ZF1 and ZF2. The population of the SCHB is shown in parentheses. Populations less than 0.5 are in red. Only populations >0.1 are included. The total SCHB population for each Cys is shown on the respective diagram. (B) SCHB distance between C7 and the C24 backbone NH and C62S and the N47 backbone NH as a function of time for trial 1. (C) Population of SCHB for the Cys correlates with their flexibility as measured by the RMSF. Standard error bars are included.

For MD of the free ERαDBD_2_ dimer, the dimer either dissociated, consistent with experimental studies or maintained a secondary structure similar to the DNA-bound dimer over the time scale of the simulation. In the two trials where the dimer remained intact, two additional OH···OC hydrogen bonding interactions between M42-S58′ and S58-M42′ (pop = 0.68), not found in the DNA-bound dimer, may have further stabilized the free dimer. Note that the stability of this dimer is likely an artifact of the simulation, which was initiated from the intact dimer Xray structure of the ERαDBD2-ERE with the DNA fragment removed. The interface is unlikely to form in solution phase, as the dimers are found to form experimentally only after the monomers are bound to the DNA.^78,79^ For these trials where the interface remained intact, the RMSF for ZF1 was similar between the monomer, the dimer, and the DNA-bound dimer. However, the RMSF for ZF2 showed a significant difference between the monomer and the dimer due to hydrogen bonding interactions between the D-box residues at the dimer interface, which contributes to decreased flexibility of the DNA-bound dimer (Figure 2C). The trial in which the dimer dissociated, was consistent with experimental evidence of a relatively weak interface,^78^ which showed no dimerization, without the ERE even with a high concentration of monomers in solution.^79^ The dimer started dissociating at ∼0.2 μs as the hydrogen bonds (C43-R55′/C43′R55, P44-T50′/P44′-T50, M44-S58′/M44′-S58) of the interface began to break, causing it to lose its integrity by 0.45 μs (SI Table S3 and Figure S3).

### Population Dynamics of the SCHBs

SCHBs to backbone N–H groups or basic residues can protect Zn-bound Cys to reduce their susceptibility to electrophiles.^19,24−26,28−31^ SCHBs are longer than those formed with O or N due to sulfur’s larger size (covalent radius = 1.03 Å)^80^ and have more diffuse electron density.^72^ The interaction energies of O/N–H···S are similar to O/N–H···O.^81,82^ SCHBs are also less linear and more perpendicular than those to O.^72^ The hydrogen bonds in a disordered protein break and reform to stabilize the protein; dynamic residues contribute to these transient H-bonding interactions.^83,84^ High variability of the H-bonds is associated with increased protein flexibility.^20^ The hydrogen bond population refers to the percentage of time during a molecular dynamics simulation where the distance between the two heavy atoms in a hydrogen-bonded pair is less than the threshold value assumed for a hydrogen bonding interaction. The number and population of SCHB interactions vary between ZF1 and ZF2 and free ERαDBD, and ERαDBD_2_-ERE. ZF1 had more than twice the total SCHB population (11.9 for ZF1 versus 5.5 for ZF2), with only three more total interactions (15 for ZF1 and 12 for ZF2). Fewer, more transient interactions shielding the ZF2 Cys is consistent with its greater reactivity with electrophiles (Figure 3A,B). For example, C7 from ZF1 forms SCHBs with A8, V9, C10, and C24, with average and total populations of 0.78 and 3.12, respectively. In contrast, C43 from ZF2 forms SCHB interactions with A45, N47, and Q48, with average and total populations of 0.24 and 0.72, indicating that it is much less electronically shielded than C7, consistent with the higher reactivity of ZF2. In addition, C24 and C62 both have 5 SCHB partners, but the more flexible ZF2 results in a lower population (1.96 vs 3.87) and more susceptibility to electrophilic attack. C59 was the most shielded of ZF2 based on its high overall SCHB population, consistent with experimental studies showing it to be less reactive than C62 in a metal-free model peptide.^27^ Overall, the negative correlation between the RMSF and the SCHB population for each Cys in free ERαDBD (Figure 3C) can be attributed to the dynamics of the protein backbone, which decreases the shielding of the Cys from electrophiles and increases reactivity.

DNA binding and dimerization protects ERαDBD_2_-ERE from oxidation relative to the free monomer.^39^ In MD simulations of the DNA-bound dimer, statistically significant increases in the SCHB populations were observed, in particular for ZF2. While the 15 SCHB partners for ZF1 remained the same with a slight population increase, the SCHB populations rose significantly for ZF2, adding a new transient interaction between C62 and R65 (pop. = 0.10, SI Table S4). The largest increases were due to the stabilization of the D-box loop upon dimerization. For example, the internal SCHB of C49 with its own backbone increased in population from 0.44 to 0.92 in the dimer alone and to 0.94 when bound to DNA. Additionally, the large increase for C43 from 0.72 in the monomeric protein to 2.8 was partly due to replacing the transient SCHB with Q48 (pop. = 0.20 in free ERαDBD) with a more populated interaction with T46, (pop. = 0.80, and a weak interaction with N47-SC (pop = 0.11) (SI Table 4). Each of these observations from the ERαDBD_2_-ERE simulations are consistent with an increase in shielding of the ZF2 Cys as a means to protect the DNA-bound dimer from electrophilic agents.

### Solvent Accessibility

**Figure 4:**
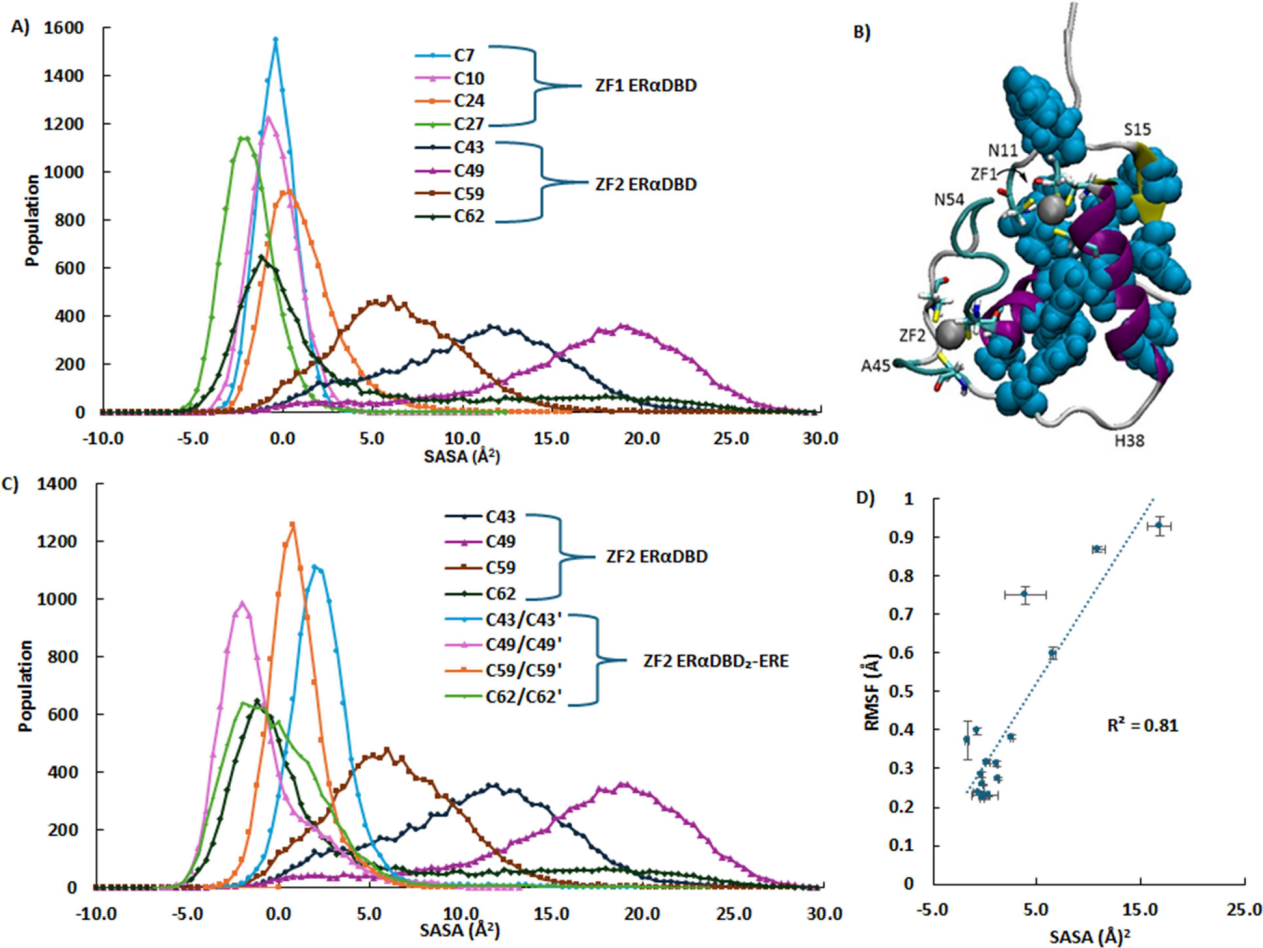
(A) SASA population distributions for the Cys sulfur centers of monomer ZF1 and ZF2. Most of ZF1 centers are buried in the protein, while those of ZF2 are more solvent-exposed. (B) ERαDBD hydrophobic core, A8, Y6, V9, Y13, Y17, Y19, V21, F30, F31, I35, Y41, A61, L64, C67, V70 and M72 modeled with VDW, contributes to the screening of ZF1. (C) Comparison of SASA population distributions for the Cys sulfur centers of ZF2 in the monomer (same as in A) and DNA bound dimer. The monomer 2 residues of ERαDBD_2_-ERE are designated with a prime (′). Dimerization decreases the solvent accessibility of the ZF2 Cys sulfurs. (D) Correlation between the Cys RMSF and the average SASA population, with standard error bars.

An analysis of structures in the PDB database found that unreactive ZFs are often buried in the core, while solvent-exposed ZFs are more reactive.^25^ SASAs were calculated for the Zn^2+^-coordinated sulfur centers of the ZF1 and ZF2 Cys in ERαDBD and ERαDBD_2_-ERE. These values reflect the atoms’ overall contribution to the surface area of the protein and are negative for buried residues.^70^ Negative SASA values are an expected result of the LCPO method when the atoms selected for analysis are not exposed to solvent.^71^ The Cys from ZF2 had greater SASA (average = 9.6 Å^2^), than those from ZF1 (average = −0.21 Å^2^) (Figure 4A,B, and SI Table S5). Three of the four Cys from ZF1, C7, C10, and C27 had negative SASAs, consistent with these unreactive residues being buried in the core.^38^ In ZF2, C43 and C49 in particular from the ZF2 D-box are significantly exposed (Figure 4B),^38^ making them an open target for electrophilic agents. Solvent accessibility also plays a prominent role in protein flexibility (Figure 4D), as the RMSF of residues increases with exposed neighbors and decreases with buried neighbors.^33,56^ In the case of ERαDBD, the residues classified as flexible that make up ZF2 leave its Cys less protected through transient SCHBs, and allow more exposure to the surrounding solvent. In contrast, while the overall SASA of ERαDBD was shown to decrease in the DNA-bound dimer,^52^ the ZF2 Cys decreased significantly (SASA average of 9.6 Å^2^ drops to 0.80 Å^2^), with the largest decrease for C49, which is adjacent to T50 at the dimer interface (Figure 4C).

The experimental study of reactivity of Cys in metal-free peptide models of ERαDBD found that C62 is more reactive than C59 from ZF2 in a truncated peptide containing these two residues.^27^ This differential reactivity was attributed to the lowering of the C62 p*K*_a_ by the adjacent basic R63, while C59 was surrounded by neutral S58 and Q60.^27^ In our simulations, R63 and C59 are found on opposite sides of the α_2_ and separated by nearly 180° such that the positive H’s of R63 are ∼13 Å from the S of C62. In contrast, our results indicate that the greater reactivity of C62 could be attributed to less shielding by SCHB interactions with the peptide backbone, which results from the greater flexibility of ZF2 in the monomer. These conclusions are in agreement with an experimental study that found ZF2 to be more susceptible to thiolate oxidation due to the loose structure of ZF2 and that DNA binding protects the thiolates from oxidation.^41^ A DFT study concluded that SCHB shielding the zinc-bound thiolate is the most essential factor in determining the reactivity of ZFP, with solvent exposure playing a lesser role. Although NH---S hydrogen bonds are shown to reduce reactivity, some unreactive ZFs do not form these bonds, implying that other factors need to be considered.^30^

## Conclusions

Electrophiles can release Zn^2+^ from the ERαDBD monomer to inhibit dimerization and DNA binding. Molecular dynamics simulations show that the differential reactivity of ZF1 and ZF2 in the free monomer and the protection from oxidation conferred by dimerization and DNA-binding can be attributed to factors related to the flexibility of the protein. Solvent accessibility can allow electrophiles access to the nucleophilic Cys while hydrogen bonding can shield the Cys thiolates by lowering their nucleophilicity. In the ERαDBD monomer, the more reactive ZF2 is conformationally flexible, contributing to a significantly lower SCHB population and greater solvent accessibility while ZF1 is buried in the protein. Dimerization occurs at the flexible D-box sequence, which becomes more rigid upon formation of the dimer interface, limiting its solvent accessibility and increasing the population of its SCHB to protect ZF2 from electrophiles. These results may also apply to other ZF proteins with differential reactivity between multiple ZF units. For example, ZF2 is more reactive than ZF1 in the C1B domain of PKCα. While both have a similar number of S–H bonds in the X-ray structure, ZF2 was more solvent-exposed, leading to greater susceptibility toward electrophilic agents.^21^ This increased understating of how the protein flexibility affects the reactivity of ZFs through modulation of solvent accessibility and shielding by SCHBs can be used to identify new inhibitors that target labile ZFs as a potential treatment for viral infections and cancer. Future studies will examine the lability of other ZFs of medicinal interest to determine how these factors affect their reactivity.

## Supporting Information Available

Root-mean-square-deviation plots, hydrogen bond donor and acceptor tables, hydrogen bond distance graphs, sulfur-containing hydrogen bond tables, and solvent accessibility tables (PDF)
