# Supplementary material for: Determination of Structural Factors Contributing to Protection of Zinc Fingers in Estrogen Receptor α through Molecular Dynamic Simulations
Source: J Phys Chem B. 2025 Feb 12;129(8):2226–34. doi: 10.1021/acs.jpcb.4c05730 (PMC11873919; doi:10.1021/acs.jpcb.4c05730)
Supplement: Supplementary file 1 — jp4c05730_si_001.pdf [file jp4c05730_si_001.pdf]

Supporting Information

Determination of Structural Factors Contributing to Protection of Zinc Fingers in Estrogen Receptor α through Molecular Dynamic Simulations

Patricia B. Lutz[a,*], Wesley R. Coombs[a], Craig A. Bayse[b]
[a]Department of Science & Mathematics, Regent University, Virginia Beach, Virginia 23464, United States
[b]Department of Chemistry and Biochemistry, Old Dominion University, Norfolk, Virginia 23529, United States
*Corresponding Author Email: plutz@regent.edu

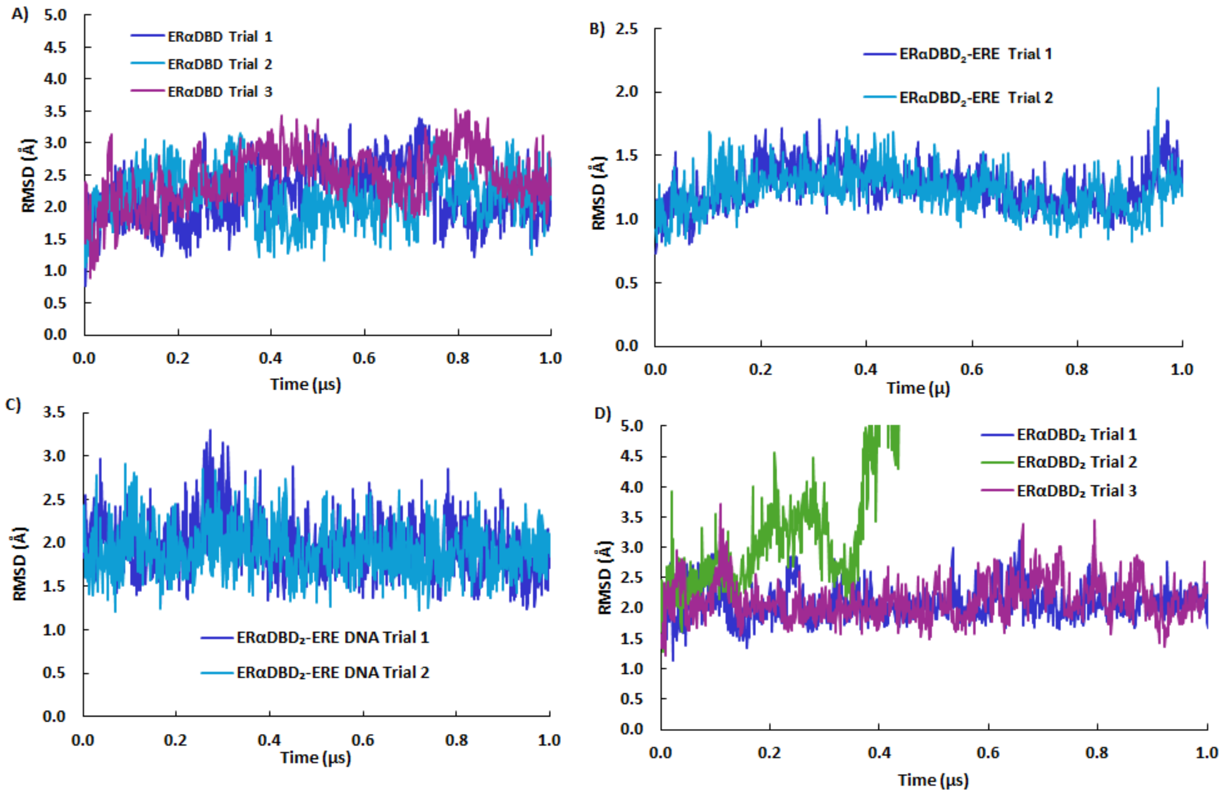

Figure S1. (A) ERαDBD Cα RMSD as a function of time. (B) ERαDBD$_2$-ERE Cα RMSD as a function of time. (C) RMSD$_2$-ERE DNA P RMSD as a function of time. (D) RMSD$_2$ Cα RMSD as a function of time.

Table S1: Hydrogen bonds at the ERαDBD$_2$-DNA. Distance D cutoff at 3.3 Å, θ cutoff at > 135°. Only populations of >0.40 are included. (Residues from monomer 1 are labeled () from 2(‘). [a]Present in the crystal structure[1]

H-Bond acceptor	H-Bond donor	population
E25$O_{\varepsilon 1/2}$	C33N_4[a]	0.98
E25’$O_{\varepsilon 1/2}$	C15N_4[a]	0.98
G31 p^{II}	R63 SC-$N_{\eta 1}$[a]	0.98
G13 p^{II}	R63' SC-$N_{\eta 1}$[a]	0.98
G31 p^{II}	R6 SC-$N_{\eta 2}$	0.97
G13 p^{II}	R63' SC-$N_{\eta 2}$	0.97
G31P^{I}	R56 BB	0.97
G13P^{I}	R56' BB	0.97
G31 p^{I}	R56 SC-$N_{\eta 2}$	0.79
G13 p^{I}	R56' SC-$N_{\eta 2}$	0.78
G4 p^{II}	Y19 SC-O_{η}[a]	0.87
G22 p^{II}	Y19' SC-O_{η}[a]	0.87

H-Bond acceptor	H-Bond donor	population
A3 p^{II}	Y19 BB[a]	0.73
A21 p^{II}	Y19' BB[a]	0.74
T30 p^{II}	R33 SC-$N_{\eta 2}$[a]	0.81
T12 p^{II}	R33' SC-$N_{\eta 2}$[a]	0.70
T30 p^{I}	Q60 SC-$N_{\varepsilon 2}$[a]	0.75
T12 p^{I}	Q60' SC-$N_{\varepsilon 2}$[a]	0.73
G13N_7	R33’ SC-$N_{\eta 1}$[a]	0.53
G31N_7	R33 SC-$N_{\eta 1}$[a]	0.58
C2OP^{II}	Y17 SC[a]	0.50
C20P^{II}	Y17’SC[a]	0.72
G4O_6	K28N_z[a]	0.88
G22 O_6	K28’N_z[a]	0.86

Table S2: Hydrogen bonds at the ERαDBD$_2$-DNA interface. Distance D cutoff at 3.3 Å, θ cutoff at > 135°. Only populations of >0.30 are included. M42 and M42’ are not considered H-bonds but are only included for comparison to ERαDBD$_2$. A diagram of the H-Bonds at the dimer interface is included. (Residues from monomer 2 are labeled with a prime (′). [a]Present in the crystal structure[1] [b]Present in previous MD studies.[2,3]

**H-Bond acceptor**	**H-Bond donor**	**population**
P44 O	T50' BB[a]	0.90
P44' O	T50 BB[a]	0.88
C43 O	R55' SC-Nε[b]	0.35
C43' O	R55 SC-Nε[b]	0.34
M42 O	S58' SC OG	0.04
M42' O	S58 SC OG	0.06

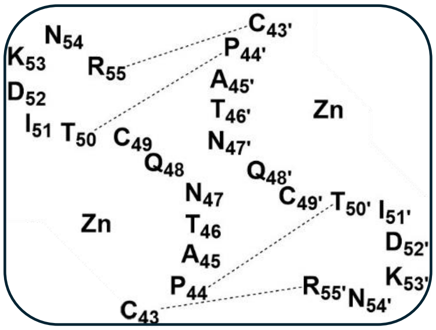

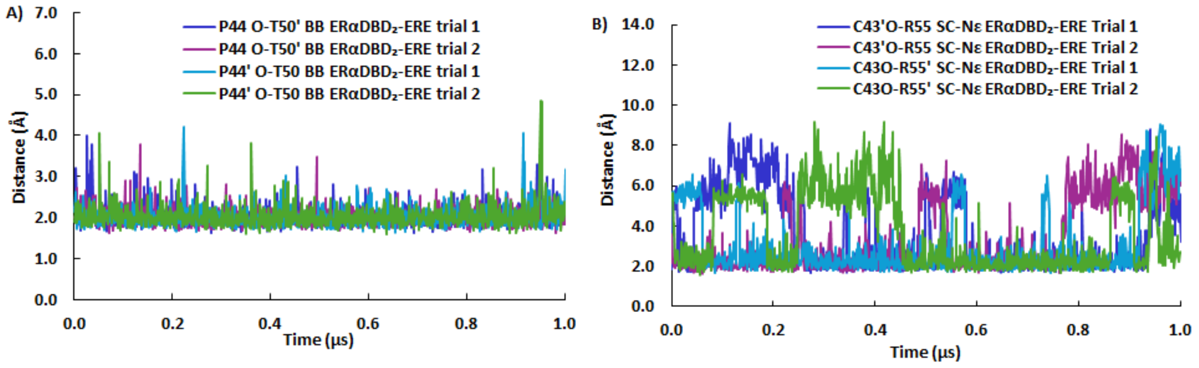

Figure S2: A)-B) Hydrogen bond distances as a function of time for the DNA-bound dimer.

Table S3: Hydrogen bonds at the $ER\alpha DBD_2$ interface. Distance D cutoff at 3.3 Å, θ cutoff at > 135°. (Residues from monomer 2 are labeled with a prime (′).

Stable $ER\alpha DBD_2$

H-Bond acceptor	H-Bond donor	pop.
P44 O	T50' BB	0.83
M42 O	S58' SC-O	0.69
C43 O	R55' SC-Nε	0.35
P44' O	T50 BB	0.81
M42' O	S58 SC-O	0.68
C43' O	R55 SC-Nε	0.30

Unstable $ER\alpha DBD_2$

H-Bond acceptor	H-Bond donor	pop.	time to break (μs)
P44 O	T50' BB	0.28	0.38
M42 O	S58' SC-O	0.28	0.38
C43 O	R55' SC-Nε	0.09	0.37
P44' O	T50 BB	0.13	0.45
M42' O	S58 SC-O	0.15	0.21
C43' O	R55 SC-Nε	0.00	0.39

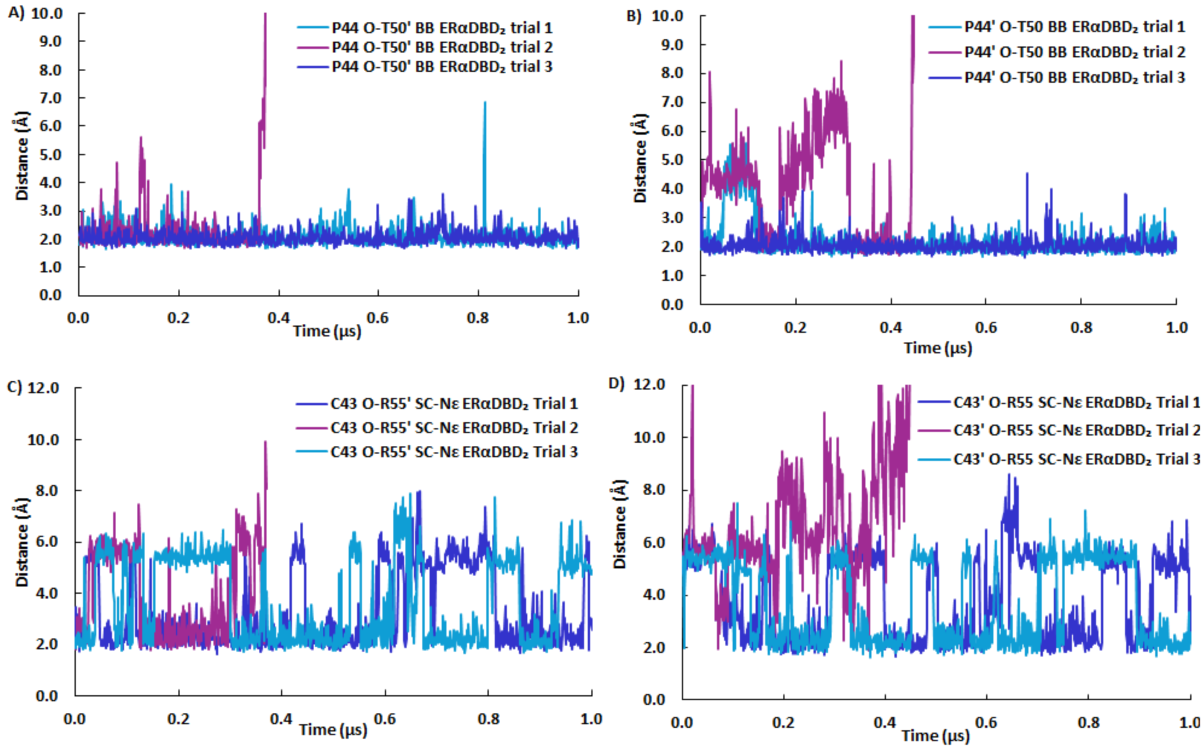

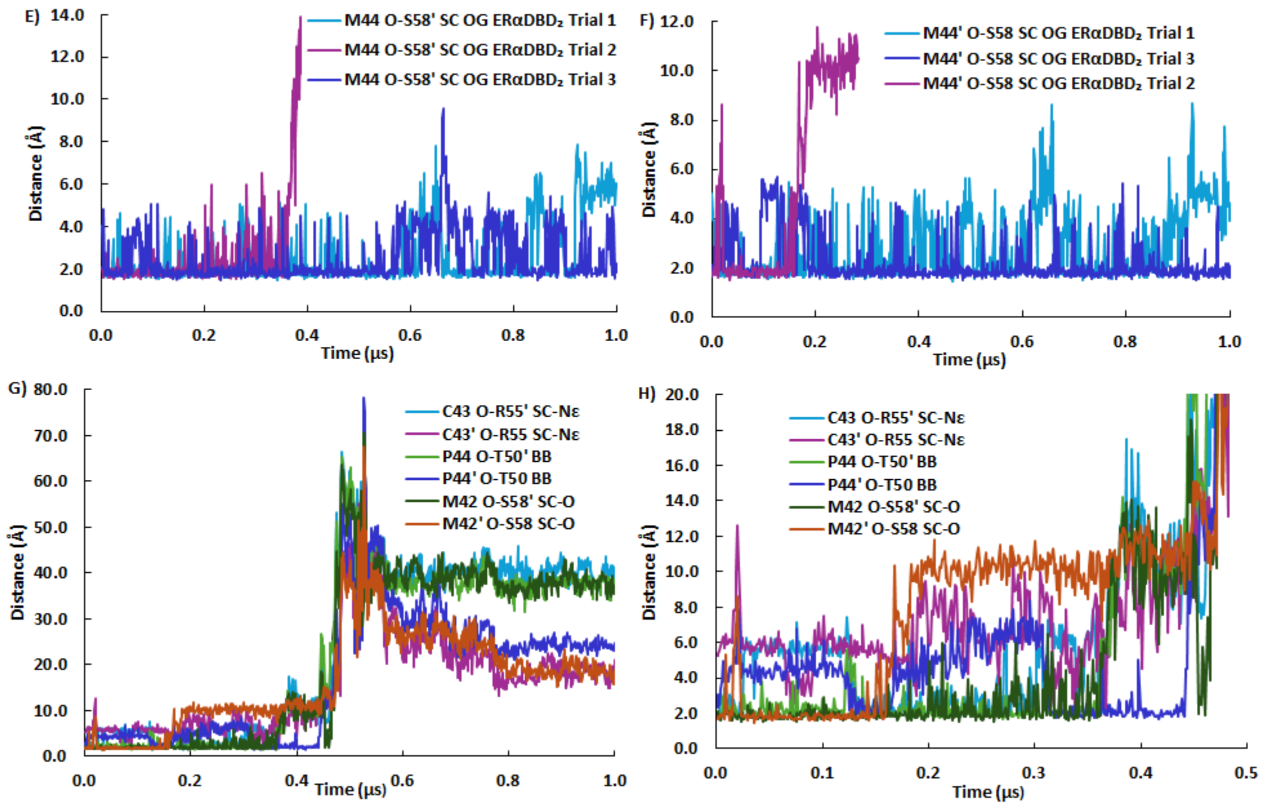

Figure S3:A)-F) Hydrogen bond distances as a function of time for the dimer alone ERαDBD$_2$ G) Combination of all H-bonds in the unstable Trial2 for ERαDBD$_2$ as a function of time. H) Magnified view of G to focus on the 0.0 – 0.5 μs time frame detailing the dissociation process.

Table S4: SCHB for each Cys for ZF1, ZF2 for ERαDBD, ERαDBD$_2$, and ERαDBD$_2$-ERE. Residues from ERαDBD$_2$ and ERαDBD$_2$-ERE monomer two are labeled (')

**ERαDBD**			**ERαDBD**		
ZF1 Cys-S	Tot. No. SCHB	Tot. SCHB Pop.	ZF2 Cys-S	Tot. No. SCHB	Tot. SCHB Pop.
C7	4	3.11	C43	3	0.718
C10	3	2.84	C49	1	0.443
C24	5	3.87	C59	3	2.33
C27	3	2.08	C62	5	1.96
**Tot.**	**15**	**11.91**	**Tot.**	**12**	**5.451**

$ER\alpha DBD_2$			$ER\alpha DBD_2$		
C7/C7'	4	3.11	C43/C43'	4	2.53
C10/C10'	3	2.85	C49/C49'	1	0.92
C24/C24'	5	3.82	C59/C59'	3	1.89
C27/C27'	3	2.19	C62/C62'	5	1.24
**Tot.**	**15**	**11.97**	**Tot.**	**13**	**6.58**
**$ER\alpha DBD_2$-ERE**			**$ER\alpha DBD_2$-ERE**		
C7/C7'	4	3.13	C43/C43'	4	2.85
C10/C10'	3	2.88	C49/C49'	1	0.94
C24/C24'	5	3.91	C59/C59'	3	1.97
C27/C27'	3	2.52	C62/C62'	6	1.55
**Tot.**	**15**	**12.44**	**Tot.**	**13**	**7.291**

Table S5: Average SASA for each Zn^{2+} bound Cys S and the total average for each ZF. for ERαDBD, $ER\alpha DBD_2$ and $ER\alpha DBD_2$-ERE.

**ERαDBD**		**ERαDBD**	
**ZF1**	**SASA (Å^2)**	**ZF2**	**SASA (Å^2)**
C7	-0.18	C43	10.9
C10	-0.29	C49	16.8
C24	1.30	C59	6.6
C27	-1.68	C62	3.9
**AVE**	**-0.21**	**AVE**	**9.6**
**$ER\alpha DBD_2$**		**$ER\alpha DBD_2$**	
**ZF1**	**SASA (Å^2)**	**ZF2**	**SASA (Å^2)**
C7/C7'	-0.20	C43/C43'	4.6
C10/C10'	-0.21	C49/C49'	-1.8
C24/C24'	1.45	C59/C59'	-0.1
C27/C27'	-2.02	C62/C62'	3.2
**AVE**	**-0.25**	**AVE**	**1.5**
**$ER\alpha DBD_2$-ERE**		**$ER\alpha DBD_2$-ERE**	
**ZF1**	**SASA (Å^2)**	**ZF2**	**SASA (Å^2)**
C7/C7'	-0.23	C43/C43'	2.6
C10/C10'	-0.59	C49/C49'	-0.7
C24/C24'	0.38	C59/C59'	1.2
C27/C27'	0.01	C62/C62'	0.3
**AVE**	**-0.11**	**AVE**	**0.8**

(1) Schwabe, J. W. R.; Chapman, L.; Finch, J. T.; Rhodes, D. The Crystal Structure of the Estrogen Receptor DNA-Binding Domain Bound to DNA: How Receptors Discriminate between Their Response Elements. *Cell* **1993**, *75* (3), 567–578. https://doi.org/10.1016/0092-8674(93)90390-C.
(2) Kosztin, D.; Bishop, T. C.; Schulten, K. Binding of the Estrogen Receptor to DNA. The Role of Waters. *Biophys. J.* **1997**, *73* (2), 557–570.
(3) Eriksson, M.; Nilsson, L. Structural and Dynamic Differences of the Estrogen Receptor DNA-Binding Domain, Binding as a Dimer and as a Monomer to DNA: Molecular Dynamics Simulation Studies. *Eur Biophys J* **1999**, *28*, 102–111.
